# Early-Stage Electrochemical Kinetics of Agave Distillates: Impact of Barrel Toasting on Polyphenol Extraction Dynamics

**DOI:** 10.3390/foods15010170

**Published:** 2026-01-04

**Authors:** Sara S. Piña-Torres, Camila S. Gómez-Navarro, Mariana García-Aceves, Marco A. Zárate-Navarro, Ana I. Zárate-Guzmán, Adriana I. Moral-Rodríguez, Francisco Carrasco-Marín, Luis A. Romero-Cano

**Affiliations:** 1 Grupo de Investigación en Materiales y Fenómenos de Superficie, Departamento de Biotecnológicas y Ambientales, Universidad Autónoma de Guadalajara, Av. Patria 1201, Zapopan 45129, Jalisco, Mexicomarco.zarate@edu.uag.mx (M.A.Z.-N.); ana.zarate@edu.uag.mx (A.I.Z.-G.); 2 UGR-Carbon, Materiales Polifuncionales Basados en Carbono, Departamento de Química Inorgánica, Facultad de Ciencias, Unidad de Excelencia Química Aplicada a Biomedicina y Medioambiente, Universidad de Granada (UEQ-UGR), 18071 Granada, Spain; amoral@ugr.es (A.I.M.-R.); fmarin@ugr.es (F.C.-M.)

**Keywords:** tequila, mezcal, raicilla, bacanora, maturation, electrochemical color index, electrokinetic

## Abstract

The maturation of distilled spirits in wooden barrels is a critical process that defines the sensory profile and quality of the final product, primarily through the release of polyphenols and flavonoids. In this study, the early extraction kinetics of these compounds in agave distillates were investigated using laboratory-scale barrels (5 L) with three toasting levels: light (185 °C/60 s), medium (210 °C/90 s), and intense (235 °C/120 s). The barrels were characterized by FTIR and Raman spectroscopy (SEM), as well as Scanning Electron Microscopy to correlate the chemical structure of the wood with the release of phenolic compounds. For this purpose, the Electrochemical Color Index (ECI), representative of polyphenols and flavonoids, was monitored daily for over 60 days. Results showed no statistically significant differences (*p* > 0.05) among the toasting levels. On the other hand, the observed kinetics exhibited four characteristic phases: (i) a linear increase during the first week due to the extraction of the most exposed compounds, (ii) a partial decrease in the second week associated with the re-adsorption of extracted compounds onto active sites remaining available on the barrel surface, (iii) a pseudo-steady state up to day 60, and finally, (iv) a subsequent linear increase. These findings provide scientific evidence supporting the official standards for the classification of aged distillates, since at least 60 days are required to condition the barrel surface to achieve a balanced extraction of polyphenols and flavonoids. The results highlight ECI as a robust and sensitive tool for monitoring the early maturation of agave distillates. Furthermore, the proposed approach not only offers complementary analytical criteria but also contributes to supporting the regulatory definitions of the reposado category, providing a practical framework for process standardization.

## 1. Introduction

The aging of distilled spirits in wooden barrels is a complex process involving chemical and physical interactions between the liquid and the barrel material, including the extraction of polyphenols, flavonoids, and other aromatic compounds that impart color, flavor, and antioxidant properties. Previous studies have analyzed extraction kinetics on a scale of several months [1,2,3], focusing mainly on *extra-aged or ultra-aged* class distillates; however, the behavior during the early stages of maturation remains poorly characterized. In the context of distillate maturation, the term ‘early stage’ typically refers to the first 60–90 days of contact with the wood, a critical period that defines the transition from silver distillate to the aged category according to regulations such as NOM-006-SCFI-2012 for tequila. While previous studies have extensively characterized the extraction of phenolic compounds over months or years, the kinetic dynamics during these first weeks remain poorly documented, particularly for agave distillates. This early time window is crucial, as it establishes the chemical basis for the subsequent sensory profile and determines the efficiency of the maturation process.

Various analytical approaches have been explored to monitor the maturation of distilled spirits, including colorimetric techniques based on absorbance indices [4], high-performance liquid chromatography (HPLC) for phenolic compounds [5], and near-infrared spectroscopy (NIR) or RAMAN as non-destructive methods [6,7]. Colorimetric indices provide rapid and low-cost measurements but are often influenced by matrix effects and lack molecular specificity. HPLC offers high selectivity and sensitivity for phenolic profiling, yet it requires extensive sample preparation and specialized instrumentation. Likewise, NIR and RAMAN provide comprehensive spectral fingerprints but depend on complex chemometric calibration models and may struggle to resolve subtle compositional changes. Nevertheless, these methods often require specialized infrastructure or present limitations in the early detection of changes, highlighting the need for alternative tools capable of capturing the subtle physicochemical transformations that occur during the initial stages of maturation. In this context, the Electrochemical Color Index (ECI) is proposed as an accessible and highly sensitive alternative for evaluating the initial evolution of maturation [8]. Given that early color formation arises from the first interactions between the spirit and the wood matrix, it becomes essential to consider the structural factors that modulate this exchange. Beyond the intrinsic composition of the spirit, extraction kinetics are strongly governed by the wood species and barrel toasting level, which modify the lignocellulosic structure, porosity, and availability of phenolic functional groups at the surface [9,10]. However, most of these studies have focused on wines and long-aged spirits, and the physicochemical characterization of wood has rarely been quantitatively related to the very early stages of maturation. Bridging this gap is particularly relevant for distillates, where short-term wood-spirit interactions play a decisive regulatory and sensory role.

Understanding the early dynamics of these compounds is essential not only to optimize industrial processes but also to scientifically support regulatory classifications. In particular, the first 60 days of maturation are critical for the definition of the *aged class* (“clase reposado”) [11], yet this time lapse has received limited scientific attention compared to long-term aging. Addressing this gap is therefore fundamental to establishing analytical criteria that link wood-spirit interactions with normative definitions.

For all the above, the present study aims to evaluate the kinetics of polyphenol and flavonoid release during the first 60 days of aging, correlating electrochemical data with the physicochemical characterization of wood. It is expected that the kinetic phases will reflect the availability of active sites in the wood, modulated by the toasting level, to clearly define the conditioning time required for the barrel surface to promote the desired sensory profile in distilled spirits. The main contribution of this study lies in its integrative approach, which combines: (i) multimodal physicochemical characterization of the wood (FTIR, Raman, SEM, and digital image texture analysis) to quantify structural changes induced by toasting; (ii) real-time electrochemical monitoring using the Electrochemical Color Index (ECI) during the first 60 days of maturation, a critical but understudied period; and (iii) the study of the correlation between structural descriptors of the wood and extraction kinetic parameters. This approach not only provides scientific evidence to support normative classifications of aged spirits but also establishes an analytical framework for the standardization of maturation processes based on objective and quantifiable criteria.

## 2. Materials and Methods

### 2.1. Barrel Design

To evaluate the impact of wood surface characteristics on extraction kinetics, 5 L oak barrels with different toasting levels were employed. For this purpose, staves of American white oak (*Quercus alba*) were subjected to a handcrafted toasting process. A propane gas torch was used to apply heat in a controlled manner. For each toasting level, the surface temperature of the wood was monitored with an infrared thermometer, and the exposure time was recorded with a stopwatch. The process was carried out by an experienced artisan from the tequila industry, who relied on the visual perception of wood color and the characteristic cracking sound to achieve the desired toasting points. The toasting levels were coded as follows: (i) Light Toast: Staves toasted to reach an average surface temperature of 185 ± 5 °C, with an exposure time of 60 ± 10 s. Visually, the surface exhibited a light brown color. (ii) Medium Toast: Staves toasted to reach an average surface temperature of 210 ± 5 °C, with an exposure time of 90 ± 15 s. The surface displayed a dark chocolate color. (iii) Intense Toast: Staves toasted to reach an average surface temperature of 235 ± 5 °C, with an exposure time of 120 ± 20 s. The surface displayed an intense darkening, and the cracking sound of the wood was clearly noticeable.

### 2.2. Barrel Characterization

The surface chemistry of barrel woods was evaluated using two complementary spectroscopic techniques to provide a comprehensive analysis of the compounds. For the characterization of functional groups and molecular vibrations, Fourier transform infrared spectroscopy with attenuated total reflectance (ATR-FTIR) was employed. Spectra were recorded in the range of 4000–400 cm^−1^ using a JASCO FT/IR-6200 spectrophotometer equipped with an ATR module. Additionally, Raman spectroscopy was used to complement the analysis, providing information on molecular vibrations and bonding modes that may be difficult to detect by infrared. Measurements were performed with a JASCO NRS-5100 micro-Raman spectrophotometer, employing a 780 nm excitation laser with a power of 4 mW. Spectra were collected in the 200–2400 cm^−1^ range.

The morphology and elemental composition of the materials were studied by Scanning Electron Microscopy using a Dual Beam Amber X microscope (TESCAN). High-resolution images were obtained in Secondary Electron Image (SEI) mode, operating at 5 keV with a working distance of 3 mm.

The surface heterogeneity of the barrel woods was evaluated by digital image analysis, which enabled objective quantification of macroscopic texture. High-resolution photographs of the staves were taken prior to barrel assembly, with standardized capture conditions to ensure data consistency. A white LED light source (45 W) with direct illumination at 95% power was used, maintaining a constant distance of 30 cm between the camera and the sample. The images were processed using a MATLAB code specifically developed for this study. To quantify heterogeneity, the Gray-Level Co-occurrence Matrix (GLCM) was calculated, from which key texture metrics were derived: Contrast, to measure pixel intensity differences; Entropy, to evaluate texture randomness; Homogeneity, to determine surface uniformity; Energy, as an indicator of pixel uniformity; and Correlation, to establish the linear relationship between adjacent pixels. This approach allowed for a detailed characterization of the surface of each barrel.

Texture analysis using the GLCM was implemented in MATLAB R2025b using the following standardized protocol: grayscale images were quantized to 256 levels. GLCM matrices were calculated for four angles (0°, 45°, 90°, 135°) with a 1-pixel offset. The matrices were symmetrized to obtain direction-invariant measurements. Texture metrics were calculated according to the following equations (Equations (1)–(4)):

Equation (1):

(1) $$\mathrm{Entropy}=-\sum_{i,j}P(i,j)\;{log}_{2}\;P(i,j)$$

Equation (2):

(2) $$\mathrm{Homogeneity}=\sum_{i,j}\frac{P(i,j)}{1+|i-j|}$$

Equation (3):

(3) $$\mathrm{Energy}=\sum_{i,j}P(i,j{)}^{2}$$

Equation (4):

(4) $$\mathrm{Correlation}=\sum_{i,j}\frac{\left(i-\mu_{i})(j-\mu_{j})P(i,j\right)}{\sigma_{i}\sigma_{j}}$$

where P(i,j) is the probability of co-occurrence of gray levels i and j, $\mu_{i}$, $\mu_{j}$ are the means, and $\sigma_{i}$, $\sigma_{j}$ are the standard deviations. The final values reported represent the average of the four directions analyzed.

### 2.3. Aging Process

The aging process was evaluated using an agave distillate with an ethanol concentration of 55% *v*/*v* as the model beverage. The initial composition of the distillate, characterized prior to aging, was as follows: aldehydes (0.827 mg/100 mL), methanol (244.61 mg/100 mL), esters (59.15 mg/100 mL), higher alcohols (279.29 mg/100 mL), and furfural (0.530 mg/100 mL). The distillate was added to the barrels, which were filled to the standard percentage commonly used in the industry to allow headspace for gas exchange. All barrels were sealed with wooden stoppers, and the aging process was carried out under controlled laboratory conditions, maintaining a temperature of 18 ± 5 °C and a relative humidity of 50 ± 5%. Table S1 compiles the experimental parameters and factors of the barrel-distilled systems studied.

### 2.4. Determination of Electrochemical Color Index (ECI)

The characterization of maturation kinetics was performed using the ECI, since it represents a robust and effective metric to jointly evaluate the polyphenolic and flavonoid fractions [12,13]. For this purpose, the methodology described by Piña-Torres et al. [8] was employed, electrochemical assays were conducted with a Biologic potentiostat/galvanostat model SP-50e using EC-Lab software (EC-Lab® V11.62.5). The electrochemical analysis of each sample was performed using Differential Pulse Voltammetry (DPV) in a three-electrode electrochemical cell (15 mL) consisting of a working electrode (glassy carbon electrode: ALS electrodes AK048 GC6X1.0, Ø = 1 mm; electroactive area 1.857 ± 0.068 mm^2^), a reference electrode (Ag/AgCl_(sat)_), and a counter electrode (platinum wire). Phosphate buffer (0.1 M, pH 4) was used as the supporting electrolyte with a cell volume of 5 mL (1:1 ratio, sample:electrolyte).

A preconcentration step was applied at 0.75 V (vs. Ag/AgCl_(sat)_) for 120 s to maximize the adsorption of phenolic compounds onto the electrode surface. Subsequently, anodic differential pulse voltammograms were recorded by scanning from −0.3 to 1.0 V (vs. Ag/AgCl_(sat)_) with the following DPV parameters: step potential = 5 mV, scan rate = 10 mV s^−1^, pulse amplitude = 50 mV, pulse width = 50 ms, and interval time = 500 ms. These operational parameters were selected based on optimal signal-to-background response as previously demonstrated. Finally, to ensure reproducibility, the working electrode was cleaned after each analysis by applying chronoamperometry at 1.2 V (vs. Ag/AgCl_(sat)_) for 120 s to oxidize adsorbed compounds, followed by ultrasonic washing with bi-distilled water and drying at room temperature (20 ± 2 °C).

The current peaks obtained in the DPV were used to calculate the ECI according to the following equation (Equation (5)):

(5) $$ECI=\;\frac{i_{0.34V}}{E_{0.34V}}+\frac{i_{0.50V}}{E_{0.50V}}$$

where ECI corresponds to Electrochemical Color Index, E_0.34V_, i_0.34V_ and E_0.50V_, i_0.50V_ correspond to the oxidation potential and current peaks of total polyphenols and flavonoids, respectively. The electrochemical color index (ECI) was conceived as a scalar descriptor that condenses the differential pulse voltammetry (DPV) response into a single meaningful parameter related to the color of aged distilled. In DPV measurements, the peak current (*i_p_*) reflects the amount and electroactivity of phenolic compounds, while the peak potential (*E_p_*) is associated with their redox thermodynamics and molecular structure. The ratio *i_p_/E_p_* therefore represents an effective electrochemical contribution that jointly accounts for both concentration and oxidation ease of the electroactive species. The summation of two peak contributions reflects the fact that distilled color arises from a mixture of phenolic and flavonoid compounds exhibiting distinct redox behaviors. Rather than identifying individual species, the ECI provides an integrated electrochemical fingerprint associated with the global chromophore composition of the beverage. Thus, the ECI is proposed as a functional index, analogous to other empirical indices used in spectroscopic and electrochemical sensor systems, aimed at correlating electrochemical response with color-related chemical features.

The working electrode was rigorously cleaned after each analysis to ensure the reproducibility of the results.

### 2.5. Statistical and Multivariate Analysis

To establish relationships between wood structural descriptors and extraction kinetic parameters, univariate and multivariate statistical analyses were performed. Bivariate correlations were evaluated using Spearman’s correlation coefficient (ρ), selected for its robustness to non-linear relationships and suitability for small datasets [14]. This analysis focused on key variable pairs identified a priori based on their theoretical relevance (e.g., A_1_ vs. Raman G, k_1_ vs. Raman G).

Multivariate analysis was conducted via Principal Component Analysis (PCA) using MATLAB R2025b (MathWorks, Natick, MA, USA). Given the exploratory nature of this study and the high dimensionality of the dataset (11 descriptive variables × 3 toasting levels), PCA was employed to reduce dimensionality, identify underlying patterns, and visualize the structure of variable relationships [15]. Prior to PCA, all variables were scaled (centered and reduced) to avoid biases due to differences in measurement units. Interpretation was based on the first two principal components (PC1 and PC2), which together explained 100% of the variance. Loadings (variable contributions) and scores (sample positions) were examined to infer structural kinetic relationships.

## 3. Results and Discussion

### 3.1. Physicochemical Characterization of Toasted Wood

**Figure 1:**
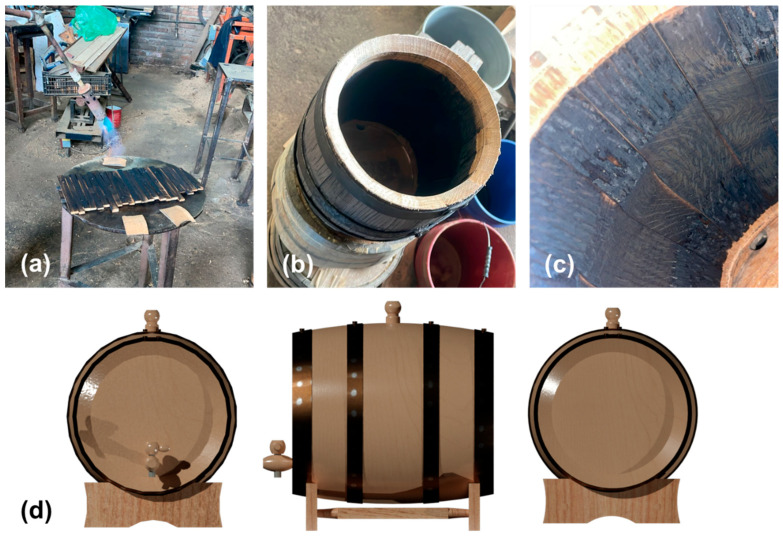
(**a**–**c**) Photographic representation of the preparation and assembly process of the barrels used in this study. (**d**) 3-D representation of the experimental setup: 5 L model barrels.

Figure 1a–c sequentially illustrate the preparation process of the oak barrels. Figure 1a shows the initial stage of stave toasting, where the controlled direct flame exposure modifies the lignocellulosic matrix of the wood. In the case of light toasting (185 °C), hemicellulose begins to decompose, releasing sugars that caramelize and contribute sweet and vanilla notes. This represents an ideal starting point to achieve delicate aromas without overpowering the distillate. For medium toasting (210 °C), lignin is significantly degraded, generating aromatic aldehydes such as vanillin (vanilla aroma) and syringaldehyde (spicy aroma). Finally, at intense toasting (235 °C), cellulose is almost completely degraded, forming higher molecular weight compounds that impart smoky, coffee, and cocoa notes [16,17,18].

Figure 1d corresponds to a representation of the experimental setup. The high surface-to-volume ratio inherent in this design (5 L) accelerates mass transfer processes, allowing the evaluation of the study objectives in a reasonable time. It is important to note that, although the kinetic rates may vary when scaling up to commercial volumes (e.g., 200 L) due to differences in surface-to-volume ratio, stave thickness, and headspace management, the fundamental wood-distillate interaction mechanisms observed in this study are applicable at larger scales.

**Figure 2:**
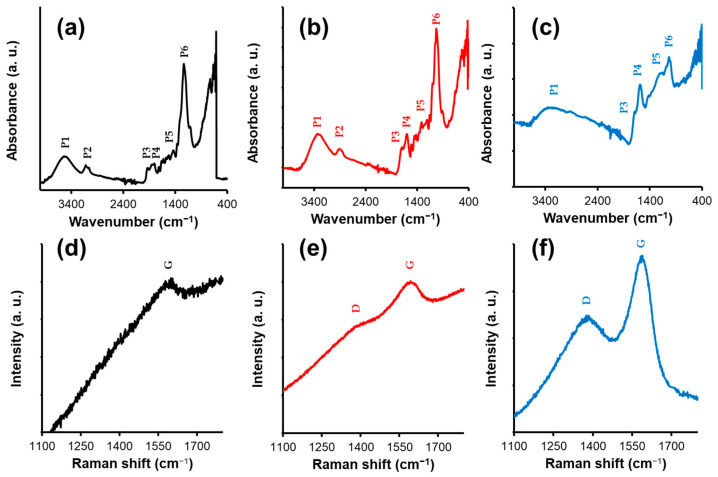
Spectroscopic characterization of thermally treated oak staves (top: FTIR; bottom: Raman): Light toast (**a**,**d**), medium toast (**b**,**e**), Intense toast (**c**,**f**).

FTIR spectroscopy characterization of thermally treated oak staves is presented in Figure 2a–c. The results show that thermal treatment not only affects surface properties but also alters the chemical composition of the wood. In the lightly toasted samples, the O–H stretching band at 3547 cm^−1^ (Peak 1, P1) and the characteristic cellulose band (Peak 6, P6) were observed, both of which progressively decrease in intensity with increasing toasting. This reduction is associated with the degradation of wood polymers, such as cellulose and hemicellulose, releasing hydroxyl groups from the network [19].

Simultaneously, intensely toasted wood shows distinctive peaks at 1666 cm^−1^ (Peak 2, P2), 1592 cm^−1^ (Peak 4, P4), and 1168 cm^−1^ (Peak 5, P5), which are associated with the formation of carbonyl groups, esters, and aromatic vibration bands, respectively. These changes are indicative of lignin degradation and pyrolysis, a key process that generates volatile compounds and increases wood porosity [20]. As toasting intensity increases, the degradation of hemicellulose and lignin exposes a higher amount of phenolic compounds and other aromatic precursors inherent to the wood matrix.

To complement these findings, Raman analysis provided additional information on molecular structure alterations (Figure 2d–f). In the lightly toasted sample, a band at 1588 cm^−1^ with an intensity of 1.14 was observed, attributed to aromatic lignin groups [21]. Medium toasting showed bands at 1383 cm^−1^ and 1588 cm^−1^ with intensities of 0.35 and 1.09, respectively. In contrast, intense toasting exhibited a marked increase in the intensity of these bands (1.78 and 4.32, respectively), suggesting greater formation of carbonized and aromatic structures. The evolution of these bands clearly indicates the degree of carbonization.

The G band (1588 cm^−1^, G) represents the stretching vibrations of sp^2^ carbon atoms in aromatic structures, while the D band (1383 cm^−1^, D) is activated by the presence of defects and structural disorder [22]. In light toasting, the exclusive presence of the G band suggests that lignin remains partially intact. In medium toasting, the appearance of the D band indicates the onset of fragmentation and defect formation. Finally, in intense toasting, the pronounced increase in intensity of both bands demonstrates extensive carbonization. The high intensity of the G band indicates massive formation of new aromatic structures, while the high intensity of the D band reveals that this new material is highly disordered, confirming a chaotic molecular structure [23].

**Figure 3:**
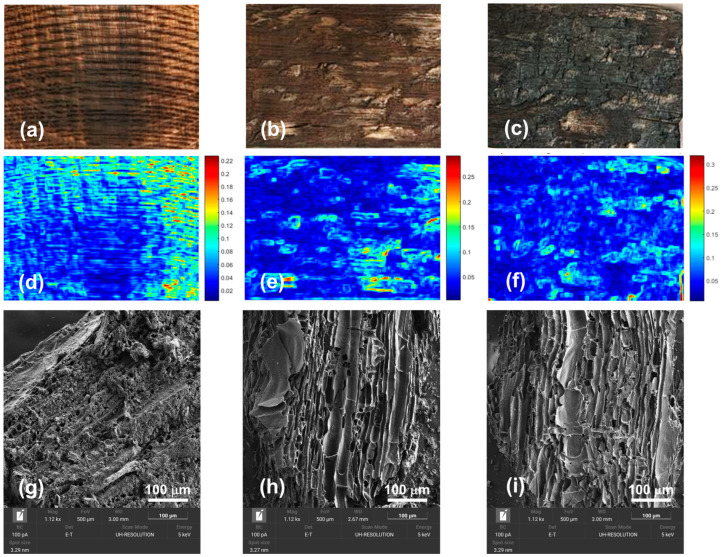
Morphological characterization of thermally treated oak staves. Digital images: (**a**) Light toast, (**b**) medium toast, (**c**) intense toast. Heterogeneity map with local standard deviation: (**d**) Light toast, (**e**) medium toast, (**f**) intense toast. SEM Micrographs: (**g**) Light toast, (**h**) medium toast, (**i**) intense toast.

To investigate the morphological alterations of thermally treated wood staves, the materials were studied by scanning electron microscopy (Figure 3). In the case of samples subjected to mild thermal treatment, the typical wood structure with interconnected hollow spaces within the individual cellulose fibers can be clearly observed [24]. In contrast, as the thermal treatment intensity increases, a noticeable contraction of the channels becomes evident, mainly due to the dehydration of the cellulose matrix and the structural rearrangement of lignin and hemicelluloses.

To complete the characterization, wood morphologies were studied through digital image analysis (Figure 3). The images show that the color of the staves changes from light brown to dark (almost black) as toasting intensity increases. The results of the image analysis confirm that wood toasting increases surface heterogeneity. As toasting intensity increased, surface heterogeneity became more pronounced, with the highest contrast and entropy values observed for intense toasting, while light and medium-toasting samples showed similar behavior (contrast: LT 252.73, MT 236.06, IT 307.06; entropy: LT 12.32, MT 11.92, IT: 12.16; homogeneity: LT 0.1824, MT: 0.2043, IT: 0.1717; correlation: LT 0.8485, MT 0.8436, IT: 0.7491). At the microscale, SEM images reveal the presence of pores, microcracks, and partially collapsed cell walls, especially under intense toasting. These morphological changes are consistent with the increase in surface heterogeneity detected by the texture analysis.

This behavior is due to the flame burning the outer layers of the wood, creating irregularities, cracks, and a more pronounced color variation, which is reflected in higher contrast values. The loss of the inherent uniformity of the original wood translates into lower homogeneity and a more random texture, evidenced by low correlation and high entropy. These trends are consistent with the visual appearance of charred surfaces, where flame-induced cracking and uneven carbonization increase macroscopic roughness and color variability. Light and medium toasting appear to exhibit similar behavior, whereas intense toasting shows a significant deviation, with much higher contrast and notably lower correlation, indicating that its surface is the roughest and least predictable.

### 3.2. Study of Maturation Kinetics

**Figure 4:**
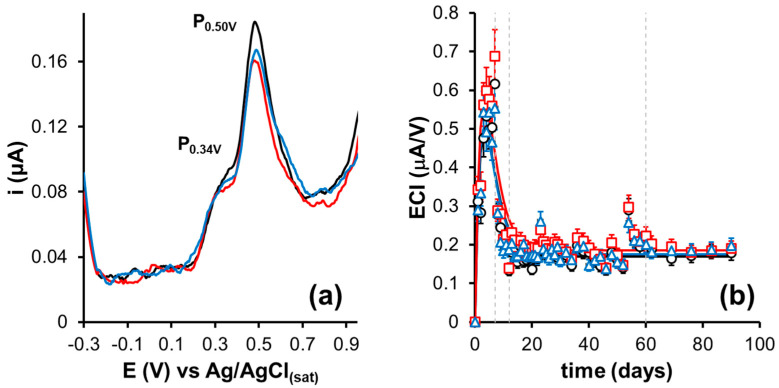
(**a**) Differential pulse voltammogram on the seventh day of aging of an agave distillate in oak barrels. Barrel toast levels: - light toast, **-** medium toast, and **-** intense toast. (**b**) Description of the maturation kinetics of the agave distillate using ECI. Barrel toast levels: **○** light toast, **□** medium toast, and **∆** intense toast. Note: In Figure 4b, error bars represent the standard deviation calculated from approximately 51 independent ECI measurements per toast level.

The electrochemical characterization of the agave distillate during the aging process was investigated using differential pulse voltammetry (DPV). The voltammetric profile of each analyzed sample is shown in Supplementary Figure S1 and Figure 4a, corresponding to the profile on day seven, where the maximum current values are observed. The voltammograms display three characteristic signals, similar to those reported in previous studies [8].

The peak P_0.34V vs. Ag/AgCl(sat)_ corresponds to the oxidation of hydroxyl groups on the A ring of the general flavonoid structure, which is associated with antioxidant activity. In contrast, the peak P_0.50V vs. Ag/AgCl(sat)_ is attributed to the oxidation of hydroxyl groups on the B ring of the main flavonoid structure. These findings are consistent with recent studies on the electrochemical color index in aged alcoholic beverages, confirming the usefulness of DPV for monitoring the evolution of bioactive compounds during maturation [12,13]. It is important to note that while these assignments, consistent with the electrochemical literature in this area, represent families of electroactive molecules rather than specific compounds, since multiple phenols and flavonoids can be oxidized across overlapping potential ranges, future studies combining HPLC-MS with electrochemistry could refine these assignments. However, for the kinetic purposes of this work (monitoring relative changes over time), this approach provides a sensitive and reproducible tool.

The extraction kinetics can be divided into four main phases (Figure 4b). During the first seven days, a linear increase in ECI values was observed, attributable to the rapid release of polyphenols and flavonoids from accessible surface sites. Between days 8 and 12, a partial linear decrease was observed, possibly related to the temporary adsorption of compounds on heterogeneously exposed active sites due to thermal treatment. From day 12 to 60, the kinetics stabilized in a pseudo-steady state, reflecting a dynamic equilibrium between extraction and adsorption. Subsequently, the indices exhibit a linear increase, associated with the release of compounds from deeper pores as the wood surface becomes conditioned and less accessible sites are reached, as previously reported in long-term maturation kinetics [1]. Results showed no statistically significant differences (F(2,150) = 0.66021; *p* = 0.51824 > 0.05) among the toasting levels, Figure S2.

To obtain fundamental insights, the extraction kinetics of phenolic compounds (polyphenols and flavonoids) during aging in thermally treated barrels were evaluated by fitting the experimental ECI data (Supplementary Table S2) to a kinetic model, Equation (6) (Figure 4b):

(6) $${ECI}_{t}={ECI}_{eq}+A_{1}e^{-k_{1}t}+A_{2}e^{-k_{2}t}$$

The proposed kinetic model describes a two-compartment system that captures the competing processes of extraction and adsorption during early maturation. In this context, A_1_ and k_1_ represent, respectively, the initial amount of extractable compounds from easily accessible surface sites and the rate constant associated with this rapid process. Similarly, A_2_ and k_2_ correspond to the fraction of compounds associated with less accessible sites (deeper pores or sites with higher interaction energy) and their slower release constant. The coefficients A_1_ and A_2_ are unconstrained, fitting parameters that may take positive or negative values, reflecting competing kinetic contributions to ECI evolution. The term ECI_eq_ represents the equilibrium value toward which the system converges after the available sites have been occupied or exhausted. This bi-exponential model is particularly well-suited to describe wood-liquid systems, where the structural heterogeneity of the lignocellulosic matrix generates multiple mass transfer pathways with different activation energies.

The model was fitted to the ECI data recorded between day 0 and day 90, corresponding to the initial increase section and the pseudo-steady state region of the curves. This model allows the description of mass transfer processes occurring during extraction. The kinetic parameters obtained for each toast level are presented in Table 1. In all cases, the regression coefficients (R) were high, confirming the suitability of the proposed model to describe the electrokinetic profiles. The rate constants (k_1_ and k_2_) did not show significant differences between treatments, indicating that toasting does not substantially affect the extraction and adsorption rates. Similarly, the equilibrium ECI (ECI_eq_) remained constant across treatments, suggesting that the final concentration of polyphenols and flavonoids does not differ significantly within the studied timeframe.

In contrast, the magnitudes A_1_ and A_2_ showed significant differences between toast levels (Table 1). A progressive decrease was observed as toasting intensity increased. In lightly toasted barrels, the extraction and adsorption amplitudes (20.848 and 20.651, respectively) were considerably higher than in heavily toasted barrels (8.187 and 7.980, respectively), while medium toasting presented intermediate values. These results can be directly related to the number of available extraction and adsorption sites. Light toasting better preserves the wood structure, allowing the release of a larger amount of compounds from the matrix. In contrast, intense toasting, although generating higher porosity, also degrades key molecular sites (e.g., –OH groups) necessary for interaction with the compounds, reducing the total amount of extractable material.

This behavior explains why medium toasting, which represents a balance between structural opening and preservation of active sites, shows a more balanced A_1_/A_2_ electrochemical contribution, suggesting a functionally favorable profile in this system, whereas intense toasting limits site availability an compromises compound extraction.

The correlation of the above information with the physicochemical characterization of the wood provide a foundation on why regulations consider the *reposado* category to reach its extraction profile in approximately 60 days, and how the trend continues toward the *aged* class.

### 3.3. Exploratory Analysis of Structural-Kinetic Patterns

To explore relationships between toasting-induced structural modifications and extraction kinetic parameters, an integrated exploratory analysis was performed using Principal Component Analysis (PCA). This multivariate technique reduces data dimensionality and identifies underlying patterns, providing a visual framework to examine variable relationships in our controlled experimental design with three ordered toasting levels [15]. Given the limited number of treatments (*n* = 3), this analysis is interpreted as descriptive and exploratory rather than inferential.

**Figure 5:**
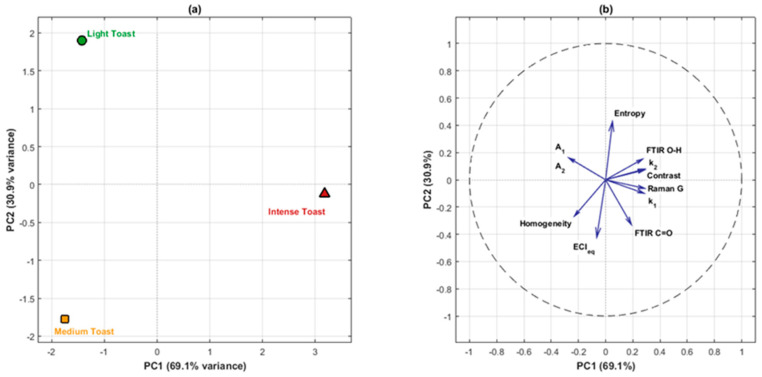
Exploratory Principal Component Analysis (PCA) of structural descriptors and kinetic parameters. (**a**) Scores plot showing the position of each toasting level in the PC1-PC2 space. (**b**) Loadings plot (correlation circle) showing variable contributions to the principal components.

Table 2 presents the loadings (variable contributions) to the first two principal components, which together explained all variance in this reduced dataset (PC1: 69.1%, PC2: 30.9%). The scores plot (Figure 5a) visually separates the three toasting levels in the PC1-PC2 space, coordinates: Light Toast (−1.43, 1.90), Medium Toast (−1.75, −1.78), and Intense Toast (3.18, −0.13). This spatial arrangement reflects the thermal treatment gradient along PC1, as expected in our controlled experimental design with three ordered conditions.

The loadings analysis (Figure 5b) reveals interpretable patterns of covariation. The PC1 axis (69.1% variance) contrasts variables associated with carbonization and roughness (Raman G: 0.359, Contrast: 0.358, k_1_: 0.353, k_2_: 0.357) against those related to extraction capacity (A_1_: −0.337, A_2_: −0.337, Homogeneity: −0.286), consistent with the progressive thermal degradation across the toasting gradient. The PC2 axis (30.9% variance) separates variables of surface structure (Entropy: 0.534, A_1_: 0.199, A_2_: 0.200) from those related to chemical composition (ECIeq: −0.528, FTIR C=O: −0.411, Homogeneity: −0.333).

To complement the PCA, Spearman rank correlation coefficients (ρ) were calculated as descriptors of monotonic trends across the three ordered toasting levels. These revealed perfect monotonic relationships: A_1_ vs. Raman G: ρ = −1.00; k_1_ vs. Raman G: ρ = +1.00; A_1_ vs. A_2_: ρ = +1.00; FTIR O-H vs. Contrast: ρ = +1.00; FTIR C=O vs. Raman G: ρ = +1.00. Given *n* = 3, these perfect correlations reflect the controlled experimental gradient rather than generalizable statistical inference, but they consistently indicate that extraction capacity (A_1_, A_2_) decreases monotonically with increasing carbonization, while initial extraction rate (k_1_) increases.

These exploratory analyses suggest that intense toasting is associated with accelerated initial kinetics but reduced total extraction capacity, whereas medium toasting occupies an intermediate position, potentially representing a balance between structural opening and preservation of active sites during the critical first 60 days of maturation. While limited by the number of experimental conditions, these patterns provide a consistent quantitative framework for understanding how structural properties of wood, modified by toasting, influence early extraction dynamics of phenolic compounds.

## 4. Conclusions

This study provides scientific evidence on the early dynamics of polyphenol and flavonoid extraction in agave distillates during the first 60 days of maturation in oak barrels with different toasting levels. The Electrochemical Color Index (ECI) proved to be a sensitive and robust tool for monitoring changes in the concentration of electroactive compounds during early maturation, revealing four characteristic kinetic phases: an initial linear increase, a partial decrease associated with re-adsorption phenomena, a pseudo-steady state, and a subsequent linear increase. Spectroscopic characterization (FTIR and Raman) demonstrated that progressive toasting promotes degradation of lignocellulosic components (cellulose, hemicellulose, and lignin), leading to increased carbonization and porosity, but simultaneously reducing the availability of functional groups required for effective interaction with phenolic compounds. Although no statistically significant differences were observed in the kinetic constants (k_1_, k_2_) among toasting levels, significant differences were detected in the extraction amplitudes (A_1_, A_2_), which were higher for light toasts and lower for dark toasts, indicating that roasting intensity primarily affects extraction capacity rather than extraction rate. Medium toasting appears to represent an optimal balance between structural openness and preservation of active adsorption sites, which supports its widespread industrial use in the production of *reposado* spirits. This study is limited by the use of 5 L barrels, a single oak species (*Quercus alba*), and a restricted maturation period of 60 days; therefore, future research should validate these findings in commercially sized barrels, include additional wood species, correlate electrochemical parameters with quantitative sensory analysis, and incorporate advanced surface characterization techniques such as atomic force microscopy to link nanotexture with extraction kinetics. Overall, the results provide objective analytical criteria for optimizing short maturation processes, reducing testing times, improving quality control in the spirits industry, and supporting regulatory classifications related to aged distillates.

## Figures and Tables

**Table 1:** Electrokinetic parameters describing the maturation process of an agave distillate in oak barrels with different toast levels.

Toasting Level	ECI_eq_	A_1_	k_1_	A_2_	k_2_	R^2^
	μA/V	μA/V	day^−1^	μA/V	Day^−1^	
Light	0.168	20.848	0.350	20.651	0.332	0.874
Medium	0.185	16.810	0.356	16.591	0.331	0.891
Intense	0.174	8.187	0.383	7.980	0.335	0.904

**Table 2:** Variable loadings in the first two principal components from exploratory PCA analysis.

Variable	PC1 (69.1%)	PC2 (30.9%)
FTIR O-H (3547 cm^−1^)	0.340	0.190
FTIR C=O (1666 cm^−1^)	0.237	−0.411
Raman G (1588 cm^−1^)	0.359	−0.079
Contrast	0.358	0.090
Entropy	0.063	0.534
Homogeneity	−0.286	−0.333
A_1_	−0.337	0.199
A_2_	−0.337	0.200
k_1_	0.353	−0.124
k_2_	0.357	0.099
ECIeq	−0.082	−0.528

Note: With only three toasting levels, PCA results are necessarily descriptive; loadings indicate directions of variation but not statistical significance.

## Data Availability

The original contributions presented in the study are included in the article/Supplementary Material, further inquiries can be directed to the corresponding author.

## Supplementary Materials

The following supporting information can be downloaded at https://www.mdpi.com/article/10.3390/foods15010170/s1: Figure S1. Evolution of the differential pulse voltammograms during the aging of an agave distillate in oak barrels with different toast levels: (a) Light toasting, (b) Medium toasting, (c) Intense toasting. Figure S2. Comparison of Electrochemical Color Index (ECI) values for agave distillates aged in oak barrels with light, medium, and intense toast levels. Statistical differences were assessed using one-way ANOVA (*p* < 0.05). Table S1. Experimental parameters and factors of the barrel-distilled system. Table S2. Evolution of the Electrochemical Color Index (ECI) during the aging of an agave distillate in oak barrels with different toast levels.
