# Supplementary material for: Early-Stage Electrochemical Kinetics of Agave Distillates: Impact of Barrel Toasting on Polyphenol Extraction Dynamics
Source: Foods. 2026 Jan 4;15(1):170. doi: 10.3390/foods15010170 (PMC12785789; doi:10.3390/foods15010170)
Supplement: Supplementary file 1 [file foods-15-00170-s001.zip › foods-4066198-supplementary.pdf]

Supplementary Materials

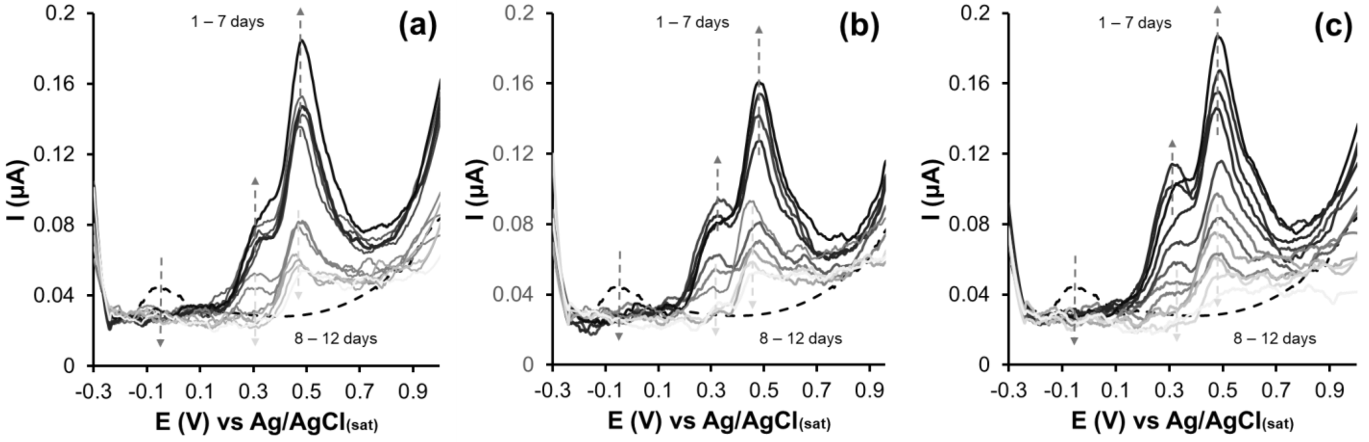

Figure S1. Evolution of the differential pulse voltammograms during the aging of an agave distillate in oak barrels with different toast levels: (a) Light toasting, (b) Medium toasting, (c) Intense toasting.

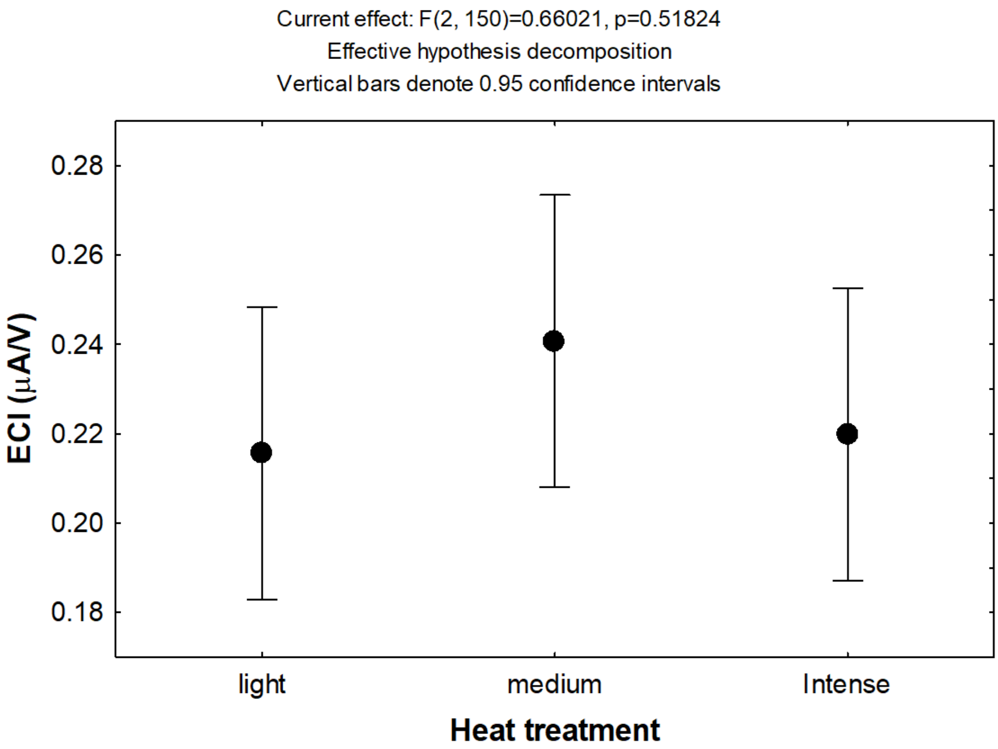

Figure S2. Comparison of Electrochemical Color Index (ECI) values for agave distillates aged in oak barrels with light, medium, and intense toast levels. Statistical differences were assessed using one-way ANOVA ($p < 0.05$).

Table S1. Experimental parameters and factors of the barrel-distilled system.

Category	Parameter	Specification
Wood	Specie	*Quercus alba* (American white oak)
	Origen	United States of America
Barrel	Capacity	5 L
	Thickness of staves	18 ± 2 mm
	Surface area to volume ratio	~0.55 cm^{-1}
toasted	Method	Propane torch, direct flame
	Temperature control	Infrared Thermometer
	Levels	Light: 185°C/60s; Medium: 210°C/90s; Intense: 235°C/120s
Distilled	Base	*Agave tequilana Weber* 100%
	Alcohol content	55% v/v
	Initial composition	See section 2.3
Maturation environment	Temperature	18 ± 5°C
	Relative humidity	50 ± 5%
	Illumination	~12h natural/artificial light, ~12h darkness
	Maturation period	90 days (daily monitoring)

Table S2. Evolution of the Electrochemical Color Index (ECI) during the aging of an agave distillate in oak barrels with different toast levels.

Time (days)	ECI (μA/V), Light toasting	ECI (μA/V), Medium toasting	ECI (μA/V), Intense toasting
0	0.000	0.000	0.000
1	0.312	0.342	0.290
2	0.283	0.352	0.333
3	0.475	0.562	0.542
4	0.533	0.598	0.491
5	0.498	0.577	0.543
6	0.504	0.559	0.466
7	0.616	0.687	0.553
8	0.278	0.289	0.282
9	0.245	0.280	0.206
10	0.204	0.214	0.184
11	0.192	0.226	0.201
12	0.133	0.139	0.187
13	0.180	0.232	0.204
14	0.188	0.212	0.168
15	0.155	0.170	0.171
16	0.195	0.210	0.183
17	0.158	0.200	0.202
18	0.162	0.179	0.172
19	0.166	0.188	0.168
20	0.137	0.179	0.173
21	0.167	0.193	0.173
22	0.164	0.191	0.167
23	0.196	0.238	0.260
24	0.190	0.208	0.189
25	0.180	0.199	0.177
26	0.177	0.191	0.165

27	0.172	0.188	0.189
28	0.191	0.206	0.175
29	0.167	0.200	0.194
30	0.183	0.189	0.159
32	0.173	0.182	0.180
34	0.147	0.163	0.161
36	0.198	0.219	0.193
38	0.183	0.209	0.197
40	0.181	0.191	0.147
42	0.159	0.176	0.160
44	0.145	0.163	0.170
46	0.136	0.139	0.138
48	0.180	0.205	0.175
50	0.162	0.173	0.155
52	0.140	0.147	0.148
54	0.290	0.298	0.258
56	0.215	0.227	0.211
58	0.194	0.208	0.210
60	0.207	0.223	0.201
62	0.189	0.202	0.182
69	0.164	0.194	0.185
76	0.171	0.180	0.184
83	0.183	0.185	0.188
90	0.178	0.190	0.197
